# Molecular Phylogeny of *Waltheria* L. (Malvaceae, Byttnerioideae) Supports the Recognition of *Stegowaltheria* as a Distinct Genus

**DOI:** 10.3390/plants15213437

**Published:** 2026-11-04

**Authors:** Thales S. Coutinho, Naédja K. M. Luna, Edlley M. Pessoa

**Affiliations:** 1 Departamento de Botânica e Ecologia, Universidade Federal de Mato Grosso, Cuiabá 78060-900, Mato Grosso, Brazil; 2 Departamento de Botânica, Universidade Federal de Pernambuco, Recife 50740-600, Pernambuco, Brazil; naedjabio@yahoo.com.br; 3 Centro de Ciências Naturais e Humanas, Universidade Federal do ABC, Santo André 09210-580, São Paulo, Brazil

**Keywords:** Hermannieae, Malvales, *Melochia*, Melochinae

## Abstract

The phylogenetic relationships of *Waltheria* (Malvaceae) species were investigated, testing its monophyly and infrageneric classification. Approximately half of the genus was sampled, including its two sections, as well as 10 species from three sections of its sister genus *Melochia*. Phylogenetic inferences were based on Bayesian inference and maximum likelihood analyses of plastid (*matK*, *trnL-F*) and nuclear (nrITS) markers. The analyses recovered *Waltheria s.l.* as non-monophyletic, with *Melochia* species nested within it. Notably, species of *W.* sect. *Stegowaltheria* form a strongly supported sister clade to *Melochia*. These results indicated the need for taxonomic changes to restore monophyly, and *Stegowaltheria* was elevated to generic rank, as this required only three new combinations, which are also provided. The new genus is recognized by clear diagnostic features, including the presence of simple trichomes on the branches, stipule shape, capsules with operculate dehiscence, and verrucose seeds. This study contributes to the systematics of Hermannieae and proposes a revised taxonomic circumscription based on molecular and morphological evidence.

## 1. Introduction

*Waltheria* L. is a genus of Malvaceae, belonging to Byttnerioideae Burnett. Its phylogenetic position in the tribe Hermannieae DC. (together with *Dicarpidium* F. Muell., *Hermannia* L., and *Melochia* L.) is strongly supported by molecular [1] and morphological data, with these genera sharing flat petals and an androecium composed of five stamens [2]. Historically, *Waltheria* and *Melochia* were grouped into the subtribe Melochinae K. Schum., especially due to the presence of an ovary with two ovules, ovoid seeds, and a straight embryo [3]. *Waltheria* can be readily distinguished, however, by its unicarpellate gynoecium, an uncommon condition within Malvaceae, whereas *Melochia* exhibits a pentacarpellate ovary.

*Waltheria* comprises approximately 60 species and is distributed mostly in the Neotropics, with some representatives occurring in Africa and Madagascar [4]. Its main centers of diversity and endemism are Brazil and Mexico, with the former (representing its primary hotspot) harboring 34 species, of which 24 are endemic [5,6]. *Waltheria* species in Brazil are especially associated with the Cerrado and Caatinga phytogeographic domains [5,6].

Schumann [7] was the first to provide a wide taxonomic study of the genus, focusing on Brazilian taxa and describing many novelties. He organized the genus into two sections, *W.* sect. *Waltheria* (as *Euwaltheria*) and *W.* sect. *Stegowaltheria* K. Schum., based on characters related to their flowers and fruits. The section *Stegowaltheria* was represented by two species, *W. bracteosa* A. St.-Hil. & Naudin and *W. macropoda* Turcz., both with flowers longer than 8 mm, plumose stigmas, and operculate capsules; the typical section included species with flowers up to 7 mm long, loculicidal capsules, and glabrous seeds. Rose [8] later described *W. operculata* Rose from Mexico and included it under *W. sect. Stegowaltheria*; he also aggregated a new diagnostic feature, the ‘great and wide’ stipules, contrasting with the ‘filiform’ stipules of the typical section.

Saunders [9] prepared a monograph of *Waltheria* that proposed a new infrageneric classification based on morphology, organizing the genus into three subgenera and two sections (in addition to several alliances and species groups), although it was never formally published. Coutinho [5] accepted Schumann’s formal classification in the taxonomic revision of Brazilian *Waltheria* and added new features for distinguishing *W.* sect. *Stegowaltheria* from *W.* sect. *Waltheria*, such as branches bearing only simple trichomes, stipules with a rounded base, and verrucose seeds (*vs.* branches with stellate trichomes, sometimes mixed with multiradiate, glandular, or simple trichomes, stipules truncate at the base, and seeds smooth or slightly crenulate at the hilum). An exception would be *W. hoehnei* J.G., described by Saunders [10] as a member of *W. sect. Waltheria* and having verrucose seeds. Coutinho [5], however, corrected this information after analyzing fresh samples of this species and stated that it bears smooth seeds.

Despite recent taxonomic advances, phylogenetic studies are generally still scarce for *Waltheria* and Hermannieae. Whitlock et al. [2] provided a phylogenetic reconstruction of Byttnerioideae using the *ndhF* plastid marker in which Hermannieae was recovered as monophyletic; *Waltheria* was represented by a single species (*W. madagascariensis* Hochr.) and was recovered as sister to other species of *Hermannia* and *Melochia*; *Dicarpidium* was not, however, sampled. Richardson et al. [11], using *ndhF* and nuclear WRKY genes, confirmed *Waltheria*’s placement within Hermannieae and Byttnerioideae, with *Waltheria* (again, only *W. madagascariensis*) nested within a clade alongside *Melochia*. Coutinho et al. [12] were the first to include several neotropical species in a phylogenetic perspective using ITS, *matK* and *ndhF* markers. Their study recovered *Melochia* (only *M. corchorifolia* L. was sampled) as sister to *Waltheria*. Few inferences on the systematics of the genus were possible, however, as only nine species of *Waltheria* were included, with only one of them from *W.* sect. *Stegowaltheria*.

More recently, in a wide phylogenomic study of Malvales and Malvaceae, Colli-Silva et al. [1] confirmed the previous data, but with *Dicarpidium* being sister to *Melochia* and *Waltheria*, with this group together being sister to the clade of *Hermannia* and *Gilesia* (although only one species of each genus was sampled). The systematics of the genera within Hermannieae therefore still require more densely sampled molecular phylogenies. Although intergeneric relationships were established by Colli-Silva et al. [1], the current circumscriptions remain to be tested for monophyly. We therefore focused here on *Waltheria*, sampling nearly 50% of that genus. Importantly, we included all of the species of *W.* sect. *Stegowaltheria*—which was crucial for assessing the infrageneric boundaries of the genus, testing the monophyly of *Waltheria* as well as Schumann’s [7] infrageneric classification, and proposing the necessary taxonomic changes.

## 2. Results

Fifty-six new sequences were generated for *Waltheria* and 13 for *Melochia*. Our complete matrix was 2448 bp long: the nrITS matrix was 545 bp long (with 22.4% being potentially parsimony-informative sites); *matK* was 806 bp long (with 3.9% potentially parsimony-informative sites); and *trnL-F* was 1097 bp long (with 2.3% potentially parsimony-informative sites) (Table S1). All sequences included in our analysis, including voucher information, are listed in Tables S2 and S3.

The nrITS tree recovered *Waltheria* s.l. as paraphyletic, in a strongly supported clade including *Melochia* species (posterior probability, PP = 1.00; bootstrap percentage, BP = 92; Figure S1) nested within it. In general, the backbone of the tree is not supported, with some exceptions, such as the strongly supported *Melochia* clade (PP = 1.00; BP = 92; Figure S1) and its relationship with the clade formed by species of *W.* sect. *Stegowaltheria* (*W. bracteosa, W. macropoda,* and *W. operculata*; PP = 1.00; BP = 99; Figure S1). Other smaller clades of *Waltheria* distributed along the tree are also strongly supported, but the relationships among them are mostly unsupported (Figure S1).

The plastid tree (*matK* and *trnL-F* combined) also recovered *Waltheria s.l.* as non-monophyletic, as the species of *Melochia* are in a weakly supported clade mixed with species of *W.* sect. *Stegowaltheria* (clade A; PP = 0.62; BP = 65; Figure S2). The internal relationships among these species are mostly unsupported, except for some smaller clades, such as that with the species of *W*. sect. *Stegowaltheria* (clade B; PP = 1.00; BP = 100; Figure S2), as well as another two clades including *Melochia* species (PP = 1.00, BP = 100; PP = 0.96, BP = 98; Figure S2). Apart from this larger mixed *Melochia–Waltheria* clade A, the other species are organized in a weakly supported clade with likewise weakly supported internal relationships. There were only two strongly supported clades: a smaller clade (clade C; PP = 0.98; BP = 98; Figure S2) and a large clade (clade D; PP = 0.97; BP = 94; Figure S2). However, the internal relationships among their species were not well supported.

The ILD test indicated some degree of incongruence between nrITS and the plastid matrices (*p* = 0.001). We nonetheless proceeded with a combined analysis because significant ILD results do not necessarily preclude data concatenation, particularly when the incongruence does not include strongly supported large clades or does not involve well-supported large topological conflicts. The main relationships contributing to the incongruence occur within the genera. In *Melochia*, examples include the positions of *M. pyramidata* L. and *M. parvifolia* Kunth. In *Waltheria*, they include the positions of *W. polyantha* K. Schum. and *W. acapulcensis* Rose, as well as the clade comprising *W. pringlei* Rose & Standl., *W. tridentata* J.G. Saunders, and *W. conzattii* Standl. The origin and evolutionary significance of these incongruences should be investigated in future studies. As these internal incongruences are beyond the scope of the present study, our taxonomic decisions were based on the combined tree, while incongruences between the datasets are also discussed.

**Figure 1:**
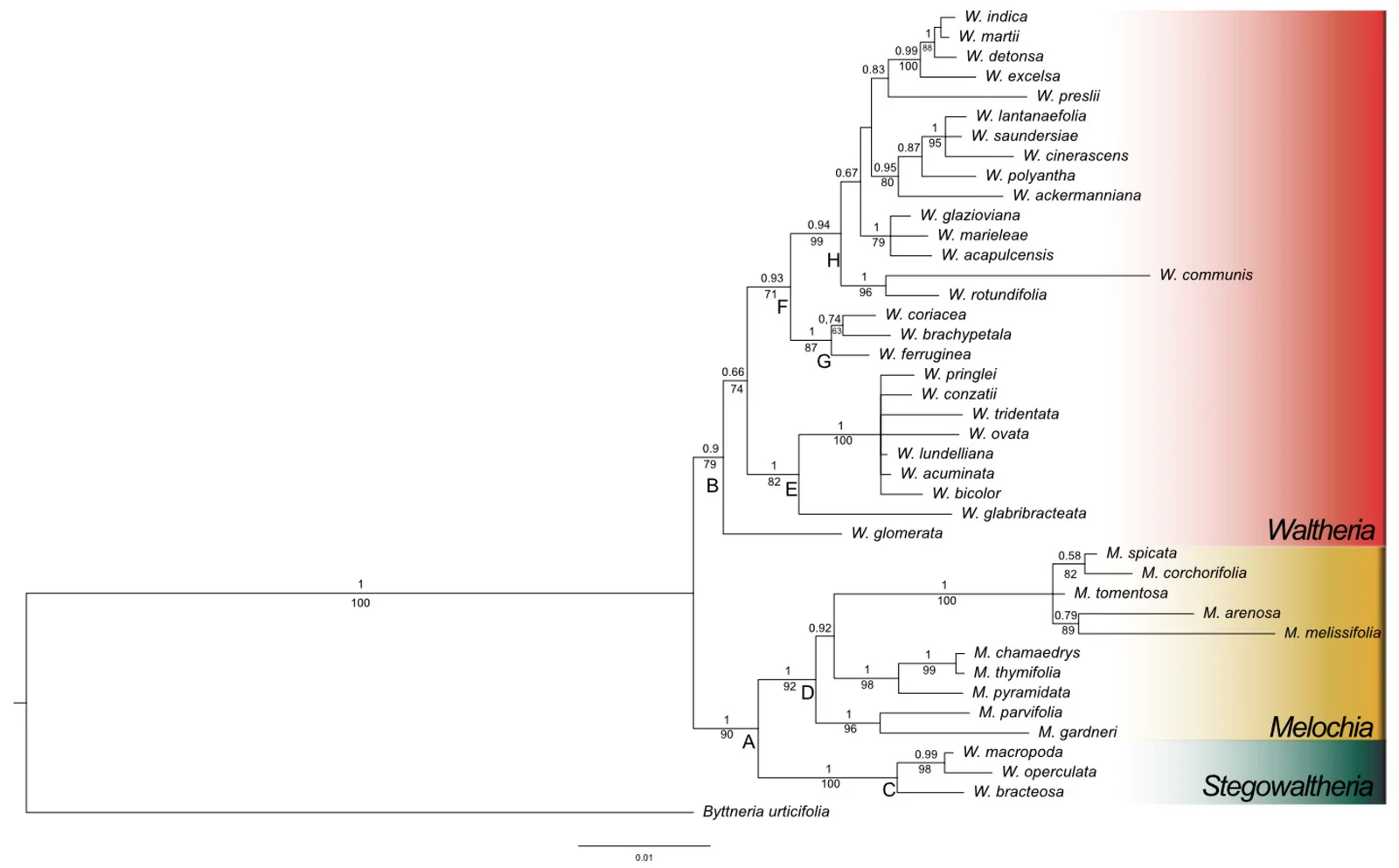
Phylogenetic relationships among *Waltheria*, *Melochia*, and *Stegowaltheria* produced using Bayesian inference of one nuclear (rITS) and two plastid regions (*matK* exon and *trnL-F* intron and spacer) (50% majority rule consensus tree). Posterior probabilities (≥0.50) are indicated above the branches, and bootstrap percentages from maximum likelihood (≥50) are indicated below the branches.

Bayesian inference (BI) and maximum likelihood (ML) analyses produced similar topologies (Figure 1 and Figure S3). Figure 1 includes both PPs from BI and BPs from ML. The combined tree (nrITS + *matK* + *trnL-F*) is considerably better resolved than each separately. It also recovered *Waltheria s.l.* as non-monophyletic (paraphyletic) since the first diverging clade (clade A, PP = 1.00; BP = 90) included all species of *Melochia* in a strongly supported clade (clade D, PP = 1.00; BP = 92) as sister to another strongly supported clade that included all species of *W.* sect. *Stegowaltheria* (clade C, PP = 1.00; BP = 100).

*Melochia* species in clade D were organized into three strongly supported clades. The first included *M. parvifolia* Kunth and *M. gardneri* Sprague (Figure 1, PP = 1.00; BP = 96), both belonging to *M.* sect. *Pyramis* Goldberg and *M.* sect. *Mougeotia* Griseb., respectively; this clade is sister to the second and third clades combined. The second clade included *M. pyramidata* L., *M. thymifolia* (C. Presl.) Goldberg and *M. chamaedrys* A. St.-Hil. (PP = 1.00; BP = 98), the former species representing *M.* sect. *Pyramis*, and the latter two representing *M.* sect. *Mougeotia*. The third clade included the type species of the genus (*M. corchorifolia* L.), along with another four species (PP = 1.00; BP = 100), four representing *M.* sect. *Melochia* and only *M. tomentosa* L. representing *M.* sect. *Pyramis*. As described, these results indicated non-monophyly of the *Melochia* sections.

The moderately supported clade B (Figure 1, PP = 0.90; BP = 79) retained only species of *W.* sect. *Waltheria*. *Waltheria glomerata* C. Presl. was found to be sister to the remaining species, which were organized into two moderate to strongly supported clades (clade E, PP = 1.00, BP = 82, and clade F, PP = 0.93, BP = 71). Clade E included *W. glabribracteata* T.S. Cout. & M. Alves as sister to the *W. ovata* Cav. complex (PP = 1.00; BP = 100) formed by seven species. The phylogenetic relationships among the species in that clade were not well recovered. Clade F is organized into two strongly supported clades (clade G, PP = 1.00, BP = 87, and clade H, PP = 0.94, BP = 99). Clade G includes *W. ferruginea* A. St.-Hil., *W. brachypetala* Turcz., and *W. coriacea* J.G. Saunders. Clade H includes the type species of *Waltheria* (*W. indica*). It is organized into four well-supported subclades, although the relationships among them were poorly recovered, and the position of *W. preslii* Walp. within one of these clades is still weakly supported.

*Waltheria* was found to be non-monophyletic in all of the trees (Figure 1 and Figures S1–S3), and there was strong support for the phylogenetic relationships between *Melochia* and *W* sect. *Stegowaltheria* as sister clades. Our data, separate or combined, provide evidence that taxonomic changes are necessary to restore the monophyly of *Waltheria*. Both *Melochia* and *Waltheria s.l.* are morphologically recognizable, and there are three possible taxonomic recircumscriptions: 1. merge the genera into a single genus; 2. elevate *W.* sect. *Stegowaltheria* to the rank of genus; or 3. merge *W.* sect. *Stegowaltheria* into *Melochia*.

## 3. Discussion

We present here the most comprehensive phylogeny of *Waltheria* published to date. Our results support its current non-monophyly, contrasting with previous molecular studies [12] and highlighting the importance of good sampling for phylogenetic inference and taxonomic decisions. Morphology-based taxonomy has long recognized the similarities between *Waltheria* and *Melochia*, with these genera sharing several morphological features, including heterostylous flowers, flat petals, and five stamens fused into a staminal tube; they differ from each other, however, in terms of the gynoecium, with the presence of one carpel in *Waltheria* but five in *Melochia* [13,14]. Our results provide the first robust phylogenetic evidence of the relationship between *Melochia* and species of *W.* sect. *Stegowaltheria* and demonstrate that taxonomic changes are required to restore the monophyly of *Waltheria*. The option of expanding *Waltheria* by including *Melochia* would create a large genus of approximately 120 species and ca. 60 necessary combinations. A second option of elevating *W.* sect. *Stegowaltheria* to the rank of genus, or a third option of merging *W.* sect. *Stegowaltheria* and *Melochia*, requires only three combinations. The second option was chosen here, as there are sets of morphological characteristics that consistently reinforce the morphological individuality of *Waltheria* and *Melochia* and distinguish the three groups.

Only 16% of the *Melochia* species were sampled in this study, and although this is the largest sample of this genus to date, few inferences are possible. *Melochia*, as circumscribed by Goldberg [14], is recovered as monophyletic, but the sections he proposed were not. Molecular and phylogenetic studies performed by Whitlock et al. [2] and Richardson et al. [11] recovered this genus as non-monophyletic, but their sampling was quite small (two and three species, respectively), making a molecular phylogenetic study focusing on *Melochia* all the more important.

**Figure 2:**
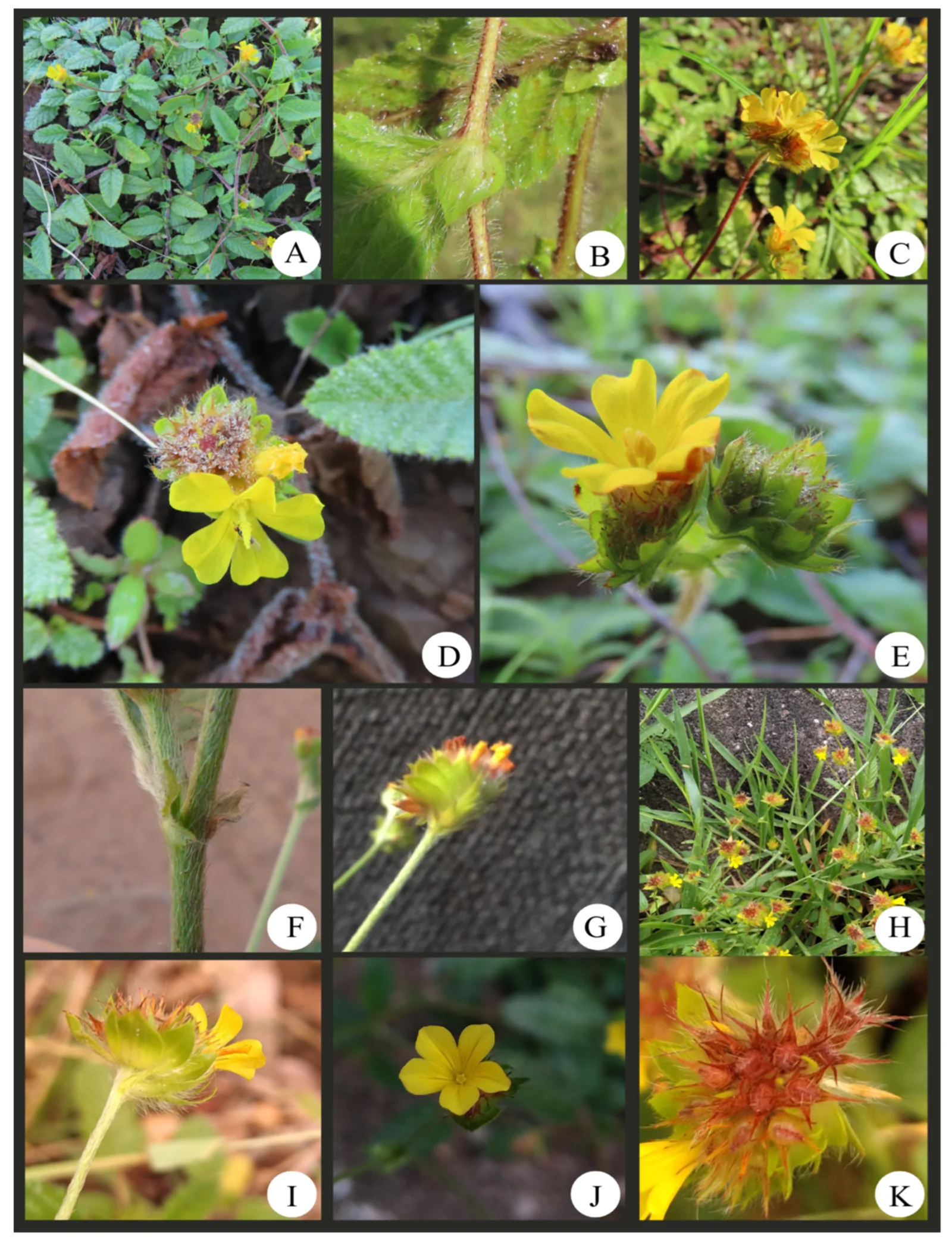
Species of *Stegowaltheria* (K. Schum.) T.S. Cout. & E. Pessoa. (**A**–**D**): *Stegowaltheria bracteosa* (**A**). St.-Hil. & Naudin) T.S. Cout. & E. Pessoa. (**A**). Prostrate habit. (**B**). Ovate and foliaceous stipules. (**C**). Inflorescence. (**D**). Longistylous flower. (**E**). Brevistylous flower. (**F**,**G**): *S. macropoda* (Turcz.) T.S. Cout. & E. Pessoa. (**F**). Lanceolate and foliaceous stipules. (**G**). Inflorescence with detail of the bracts and bracteoles. (**H**–**K**): *S. operculata* (Rose) T.S. Cout. & E. Pessoa. (**H**). Habit. (**I**). Inflorescence with detail of the bracts and bracteoles. (**J**). Homostylous flower. (**K**). Infructescence with capsules enclosed by a persistent calyx. Images: (**A**–**E**). I.L. Moraes.

Our choice of preserving the morphological identity of the genera within Hermannieae (Table 1) motivated us to not include *W.* sect. *Stegowaltheria* under *Melochia*. This clade differs from *Melochia* by having a gynoecium 1-carpellate, stigma penicillate, a 1-carpellate and obpyramidal capsule with operculate dehiscence, and verrucose seeds (*vs.* 5-carpellate, clavate stigmas, globoid 5-cocca or pyramidal pentapterous capsules with septicidal or loculicidal dehiscence and seeds smooth or with arista or winged) [5,14]. The new genus, *Stegowaltheria* (K. Schum.) T.S. Cout. & E. Pessoa (Figure 2), can be distinguished from *Waltheria* by branches and leaves with only simple trichomes, foliaceous, and wide stipules with a rounded base, capsules with operculate dehiscence, and verrucose seeds (*vs.* branches and leaves with predominantly stellate trichomes, scarious stipules with a truncate base, capsules with loculicidal dehiscence, and smooth seeds with or without a crenulate hilum) [5].

The phylogenetic relationships among the *Stegowaltheria* species were well resolved, with *W. bracteosa* being sister to the other two species. It is distinguished by having a prostrate habit (*vs.* erect to semi-erect), hirsute branches (*vs*. sericeous), and wider and ovate stipules (*vs.* narrower and lanceolate) [5]. *Waltheria macropoda* and *W. operculata* are sisters and share many morphological characteristics, which sometimes makes distinguishing between them very difficult [9].

Among the species that remained in *Waltheria s.s.* (clade B), *W. glomerata* was found to be sister to the rest. It is notable for having white petals, leaves with actinodromous-reticulate venation, and a shrubby habit exceeding two meters tall; it shares only the vein pattern with *W. involucrata* Benth. and *W. berteroi* (Spreng.) J.G. Saunders, neither of which was sampled in this study [9]. The other species are organized into two clades. In the smaller one (clade E), the South American *W. glabribracteata* is sister to the *W. ovata* complex (seven species sampled), a group composed mostly of Mexican species, except for *W. ovata*, which is endemic to South America [4,15]. A multidisciplinary approach combining morphometric and genomic data will be necessary to resolve the taxonomy of the *W. ovata* complex. The low genetic variation observed in this clade, as compared with the others, is consistent with either recent divergence or ongoing gene flow among its component species. Our molecular data do not allow us to distinguish between these two processes; further analyses focusing specifically on this complex are therefore needed to test these hypotheses [16]. *Waltheria glabribracteata* is not part of the complex, as it differs in many aspects, including a staminal tube totally fused in longistylous flowers and an elongate-plumose stigma (*vs.* filaments not totally fused and clavate or filiform stigmas) [4,15].

The larger clade F, predominantly Brazilian (except for *Waltheria detonsa* A. Gray, *W. preslii* Walp. and *W. acapulcensis* Rose, which are Mexican species), is subdivided in two subclades. Subclade G includes only three species (*W. brachypetala*, *W. coriacea*, and *W. ferruginea*), all characterized by the presence of multiradiate and stipitate trichomes on the branches, clavate stigma, and capsules with a rounded apex [5]. Six other species of *Waltheria*, not sampled here, have the same homogeneous set of characters, and all are endemic to Brazil (mostly habiting areas of Cerrado and campos rupestres) [6,10,17]. All of the species in subclade H have branches predominantly recovered by stellate and sessile trichomes, a penicillate stigma of the elongate-plumose or rounded-plumose type, and capsules with a truncate apex [4,5]. The first diverging species, *W. communis* A. St.-Hil. and *W. rotundifolia* Schrank, have an unusual pattern of capsule opening, with dehiscence lines flanking the entire surface of the fruit, causing it to open completely, and the pericarp releasing two valves at the end. This opening pattern was called total dehiscence by Coutinho [5] and Saunders [9], and it is also observed in *W. vernonioides* R.E. Fries, which was not sampled here. In contrast, the remaining species in this subclade have partial dehiscence, with the suture starting at one of the edges of the apex and then crossing it towards the base, but ending there.

### Taxonomic Treatment

***Stegowaltheria*** (K. Schum.) T.S. Cout. & E. Pessoa, ***stat. nov.***

Type species: *Stegowaltheria bracteosa* (St.-Hil. & Naudin) T.S. Cout. & E. Pessoa. (≡ *Waltheria bracteosa* A. St.-Hil. & Naudin, Ann. Sci. Nat., Bot., sér. 2, 18: 37. 1842.

≡ *Waltheria sect. Stegowaltheria* K. Schum., Fl. Bras. 12(3): 52. 1886.

Diagnosis: *Stegowatheria* is similar to *Waltheria* in sharing heterostylous flowers, gynoecium with 1 carpel, stigma penicillate, and fruit type capsule. They differ by branches and leaves with only simple trichomes (*vs.* trichomes always stellate, mixed with simple, multiradiate, 2−3-radiate and glandular ones, or not, in *Waltheria*), stipules foliaceous and basally rounded (*vs.* scarious and truncate), capsules operculate (*vs.* loculicidal or bivalvate), and seeds verrucose (*vs.* smooth or slightly crenulate).

Description: Subshrubs erect or prostrate. Branches erect or decumbent, hirsute or sericeous, trichomes only simple, hyaline; stipules foliaceous, base rounded, apex acuminate, veins 3–4. Leaves simple, spirally alternate; leaf blade green, hirsute to sericeous, only simple trichomes on both surfaces; venation actinodromous. Inflorescence long-peduncled, glomerule apical, bracts densely disposed. Flowers heterostylous, sessile, disposed in pairs and surrounded by foliaceous bracteoles. Calyx gamosepalous, tubular-campanulate, lobes free, acuminate. Corolla dialipetalous, gold-yellow, petals spatulate, adnate to the staminal tube basally. Stamens 5, forming a staminal tube, glabrous, free filaments present (in brevistylous flowers) or absent (homostylous or longistylous flowers). Carpel 1, style straight, pubescent, stigma penicillate, elongate-plumose. Capsule obpyramidal, membranaceous, dehiscence operculate; seed 1, black, obovoid, glabrous, verrucose.

**Figure 3:**
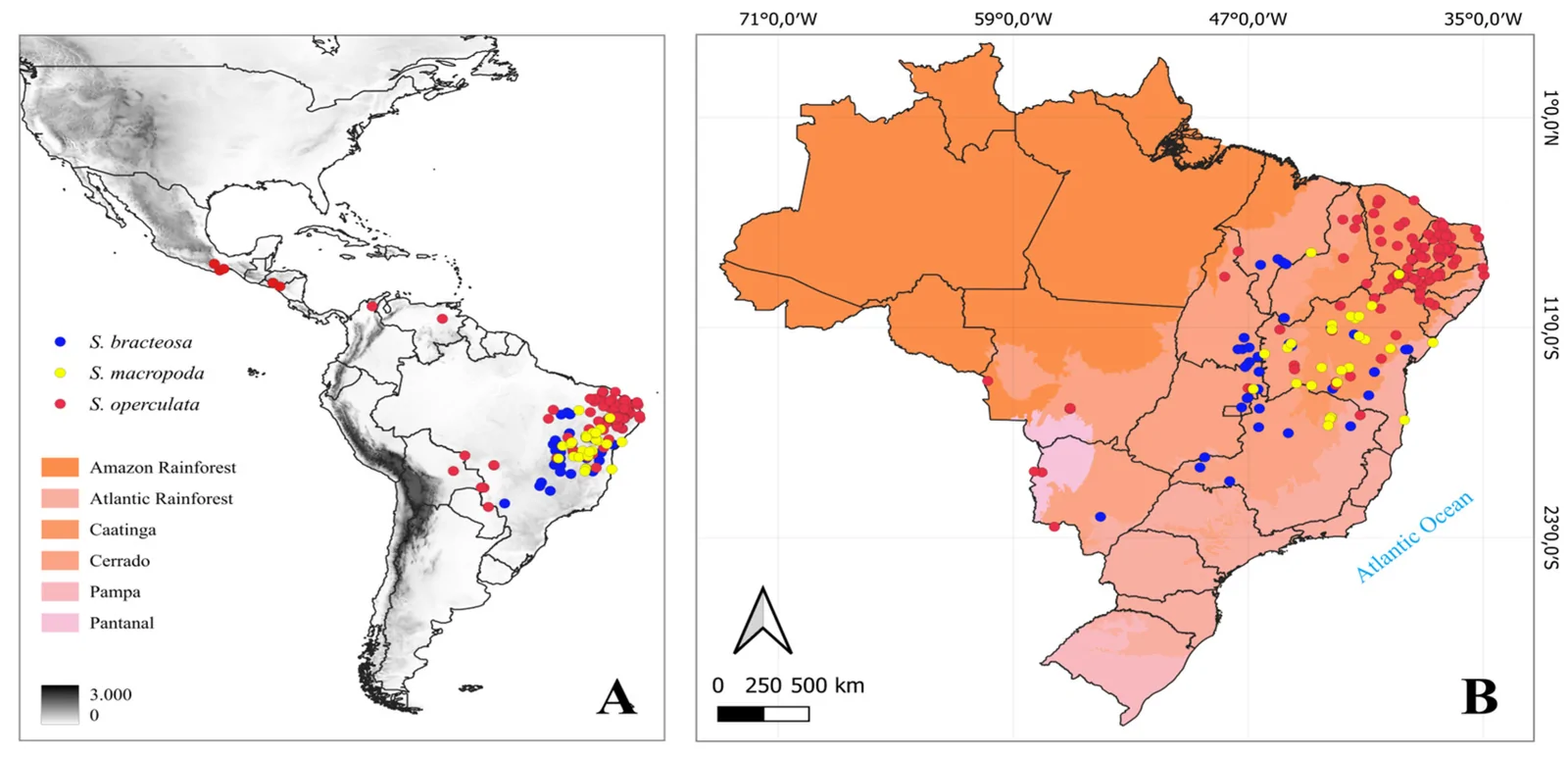
Geographic distribution map of *Stegowaltheria* (K. Schum.) T.S. Cout. & E. Pessoa species. (**A**). Geographic distribution in the Americas, with *Stegowaltheria operculata* being the most widely distributed species. (**B**). Main center of diversity and endemism in Brazil.

Geographic distribution and conservation: *Stegowaltheria* comprises three species distributed from Mexico to Brazil, Bolivia, and Paraguay [4,5,6]. *Stegowaltheria operculata* is the most widely distributed species, occurring in North America (Mexico), Central America (El Salvador and Honduras), and South America (Brazil, Bolivia, Colombia, Paraguay, and Venezuela) [4,6], whereas *S. bracteosa* and *S. macropoda* are endemic to Brazil [5,6] (Figure 3). In Brazil, the primary center of diversity and endemism of the species is dry Caatinga and Cerrado habitats [5,6]. According to Coutinho [5], all species of *Stegowaltheria* (as *W.* sect. *Stegowaltheria*) are classified as Least Concern, especially due to their extensive geographic distributions.

***Stegowaltheria bracteosa*** (St.-Hil. & Naudin) T.S. Cout. & E. Pessoa, comb. nov. ≡ *Waltheria bracteosa* St.-Hil. & Naudin, Ann. Sci. Nat., Bot., sér. 2, 18: 37. 1842.

Type: —BRAZIL. Goyaz [Goiás]: s.l., 1841, *G. Gardner 3607* (holotype: G G00358710 [image!]; isotypes: E E00012597 [image!], F F0073609F and F0073610F [images!], G00358711 and G00358712 [images!], GH GH00057000 [image!], K K000380968 [image!], NY NY00074097 and NY00074074 [images!], P P02273692 and P02273693 [image!], W W0070921 [image!]).

***Stegowaltheria macropoda*** (Turcz.) T.S. Cout. & E. Pessoa, comb. nov. ≡ *Waltheria macropoda* Turcz., Bull. Soc. Imp. Naturalistes Moscou 31(1): 216. 1858.

Type—BRAZIL. Bahia: La Serra Jacobina, s.d., fl., *J.S. Blanchet 2579* (lectotype, designated by Saunders & Dorr: KW KW001000116 [image!]; isolectotypes: E E00012601 [image!], F F0073624F [image!], G G00358724 [image!], LE LE00006874 and LE00006875 [images!], P P02273713 and P02273714 [images!], W W0071727 [image!]). [Remaining syntypes: Gardner s.n.]. Non *Waltheria macropoda* Klotzsch ex Schomburgk, nom. nud.

***Stegowaltheria operculata*** (Rose) T.S. Cout. & E. Pessoa, comb. nov. ≡ *Waltheria operculata* Rose, Contr. U.S. Natl. Herb. 5: 183. 1899.

Type—MEXICO. Between Tapana, state of Oaxaca, and Tonala, state of Chiapas, 200 to 500 feet, 1 to 3 August 1895, fl., fr., Nelson 2876 (holotype: US US00479029 [image]!; isotypes: GH GH00056991 and GH00056992 [images]!).

***Waltheria*** L.

Type species: *Waltheria indica* L., Sp. Pl. 2: 673. 1753.

=*Astropus* Spreng., Neue Entdeck. Pflanzenk. 3: 64. 1822.

=*Lophanthus* J.R. Forst. & G. Forst. in Char. Gen. Pl., ed. 2.: 27 (1776), nom. illeg. homonym. post.

=*Sitella* L.H. Bailey, Gentes Herbarium 4: 349. 1940.

Description: Shrubs to subshrubs, erect, rarely prostrate. Branches with trichomes predominantly stellate, mixed or not with simple, 2-3-rad, multiradiate or glandular, sessile or stipitate; stipules scarious, base truncate, apex acute or acuminate, veins 1 or absent. Leaves with trichomes similar to the branches; venation actinodromous. Flowers heterostylous, sessile (rarely pedicel, c. 1 mm long), disposed in pairs and surrounded by bracteoles. Carpel 1, style lateral, geniculate at base, pubescent, stigma penicillate, round-plumose, elongate-plumose, clavate, or rarely filiform. Capsule obpyramidal or obovoid, membranaceous throughout, or from the median region to the base, apex truncate or rounded, with loculicidal dehiscence; seed 1, black, obovoid, glabrous, totally smooth or slightly crenulate on the hilum.

Geographic distribution and conservation: The genus currently comprises approximately 57 species, primarily distributed in the Neotropics, with few species occurring in Paleotropical regions [5,9]. Brazil is the primary center of diversity and endemism, with 34 species, 24 of which are endemic, distributed especially across the Cerrado phytophysiognomy [5,6]. Although well represented in this biome, significant numbers are also recorded in the Caatinga (16 spp.) and the Atlantic Forest (14 spp.) [5,6]. According to Coutinho [5], approximately 14 species can be classified as experiencing some degree of threat, some because they are known from only a few populations and others because of habitat loss, such as in *Restingas*.

Identification Key to the Hermannieae genera (Byttnerioideae, Malvaceae)

1. Gynoecium 1−2-carpellate ……………………………………………….……….........….... 2
2. Flowers not heterostylous; gynoecium 2-carpellate, 2-loculate …………………………………………………………………………...… *Dicarpidium* F. Muell.
2′. Flowers heterostylous; gynoecium 1-carpellate, 1-loculate ………………………..…….. 3
3. Branches with only simple trichomes; stipules foliaceous; capsule operculate; seeds verrucose ..……………...………………..…….. *Stegowaltheria* (K.Schum.) T.S. Cout. & E. Pessoa
3′. Branches with predominantly stellate trichomes, mixed or not with multiradiate, simple, glandular trichomes; stipules scarious; capsule loculicidal; seed smooth, sometimes slightly crenulate at the hilum ………………………………………………..…..... *Waltheria* L.
1′. Gynoecium 5-carpellate ………..….…………………..…...................................................... 4
4. Flowers not heterostylous; filaments usually free; ovary with 3–many ovules per locule …..…………………………………………..…………………………………….... *Hermannia* L.
4′. Flowers heterostylous; filaments fused into a staminal tube; ovary with 1−2 ovules per locule …..…………………………………...………………………..…....................... *Melochia* L.

## 4. Materials and Methods

### 4.1. Taxon Sampling

This study included 30 species of *Waltheria*, totaling 50% of the genus; three belong to *W.* sect. *Stegowaltheria*, and 27 belong to *W.* sect. *Waltheria sensu* Schumann [7]. This represents the largest sampling of the genus in a phylogenetic study to date. As confirmed by Colli-Silva et al. [1], *Melochia* is its sister genus, so we sampled ten species of this genus belonging to three of the five sections *sensu* Goldberg [14] (three *M.* sect. *Mougeotia* Griseb.; three *M.* sect. *Pyramis* Goldberg; and four *M.* sect. *Melochia* L.). *Byttneria urticifolia* K. Schum. (Byttnerieae–Byttnerioideae) was used as the outgroup. Most specimens were collected in the field (21 in total) and preserved in silica gel for DNA extraction. Herbarium material for 11 species was also used, as we were not able to collect them in the field. Vouchers were deposited in the CEN, HCDAL, HUEFS, MEXU, SPF, UB, UFP and UFMT herbaria. Vouchers for all specimens analyzed are presented in Table S2.

### 4.2. DNA Extraction, Amplification and Sequencing

Total DNA was extracted from 25 mg of silica-gel-dried tissue using the 2 × CTAB method [18]. Three molecular markers were selected based on their previous application in phylogenetic studies of *Waltheria*: the plastid markers *matK* and *trnL-F* (*trnL* intron + *trnL-F* intergenic spacer) and the nuclear ribosomal ITS (nrITS: ITS1 spacer +5.8S gene +ITS2 spacer). Amplification was not successful for *ndhF* and ETS for most species, which are therefore not included here. Despite some ITS limitations regarding hybridization scenarios and incomplete lineage sorting [16], it is widely used in phylogenetic studies because of its high variability. A partial copy of *matK* was amplified using the primers 390F, 1326R, trnK2R, and 3914F [19,20]. The primers C and F from Taberlet et al. [21] were used for the *trnL-F* spacer/intron region. The primers described by Sun et al. [22] were used for ITS. All amplifications used a final volume of 25 μL: 12.5 μL of Dream TAq mix (Thermofisher, Waltham, MA, USA), 0.5 μL of forward primer, 0.5 μL of reverse primer, 10.5 μL of H_2_O, and 1 μL of total DNA. The *matK* sequence used involved: 94 °C for 1 min; 35 cycles of 94 °C for 30 s, 48 °C for 40 s, and 72 °C for 40 s, followed by a final extension at 72 °C for 5 min. The *trnL-F* sequence used involved: 94 °C for 1 min; 35 cycles of 94 °C for 30 s, 53 °C for 40 s, and 72 °C for 40 s, followed by a final extension at 72 °C for 5 min. The nrITS sequence used involved: 94 °C for 2 min; 30 cycles of 94 °C for 1 min, 48 °C for 1 min, and 72 °C for 1.5 min, followed by a final extension at 72 °C for 4 min. The PCR products were then purified using an AMpure XP type kit (Beckman Coulter, Brea, CA, USA) and sequenced with a BigDye^®^ Terminator v. 3.1 Cycle Sequencing kit (Applied Biosystems, Waltham, MA, USA), following the manufacturer’s protocols. Sequencing was performed with an ABI3730xl Genetic Analyzer (Applied Biosystems, Waltham, MA, USA).

### 4.3. Alignment and Phylogenetic Analysis

Forward and reverse chromatograms were combined using Geneious Prime v.2021.1.1 (Biomatters, Auckland, New Zealand). The sequences generated in this study are available in GenBank, and their accession numbers are listed in Table S3. We proceeded with alignment using the MUSCLE [23] plugin implemented on Geneious. We checked gene tree incongruence using the incongruence length difference test (ILD, [24]) in PAUP* v.4.0a159 [25] with 1000 replicates. Substitution models for each region were selected using JModelTest v.2.1.10 [26] using the Bayesian information criterion (Table S1). For Bayesian inference (BI), we used MrBayes v.3.2.7a [27] (implemented at the CIPRES Science Gateway portal [28]) on separate nrITS and combined plastid regions (*matK* + *trnLF*), followed by a combined dataset (nrITS + *matK* + *trnLF*). We employed GTR + G for all regions, in two independent runs with four chains each, with Markov chain Monte Carlo (MCMC) parameters defined for 20,000,000 generations, sampling every 2000 trees. The first 2500 trees were discarded as burn-ins (25%). Convergence between the runs was verified using Tracer v.1.6, looking for ESS values above 200 [29]. Maximum likelihood (ML) was performed using RAxML v.8.1.20 [30], also implemented at the CIPRES Science Gateway portal, with 1000 pseudo-replicates of thorough bootstrap using the same models cited above. All trees were edited using FigTree v1.4.4 software [31]. Clades with ≥0.95 posterior probability (PP) were considered strongly supported; those between 0.94 and 0.85 were classified as moderately supported. Clades with bootstrap percentages (BPs) ≥ 85% were considered strongly supported, and those between 0.84 and 0.70 were considered moderately supported [32,33,34].

## Figures and Tables

**Table 1:** Morphological comparisons among the genera of Hermannieae DC.

Genera/Characters	*Dicarpidium*	*Hermannia*	*Melochia*	*Stegowaltheria*	*Waltheria*
**No. species**	1 sp.	c. 170 spp.	c. 63 spp.	3 spp.	c. 58
**Stamen** **arrangement**	Staminal tube	Staminal tube	Staminal tube	Staminal tube	Staminal tube
**No. carpels**	2	5	5	1	1
**No. styles**	Unknown	5	5	1	1
**Stigma type**	Unknown	Clavate	Clavate	Penicillate (elongate-plumose)	Penicillate (elongate-plumose or round-plumose), clavate, or linear
**Capsule shape**	Unknown	Rounded, angled or horned	Globoid 5-cocca or pyramidal pentapterous	Obpyramidal	Obpyramidal or obovoid
**Capsule dehiscence**	Unknown	Loculicidal	Septicidal, loculicidal, septicidal–loculicidal	Operculate	Loculicidal partial or total
**Seed shape**	Unknown	Reniform, sub-reniform, comma-shaped, kidney-shaped, or C-shaped	Ovoid or trigonous	Obovoid	Obovoid
**Seed(s) per locule**	Probably 1–2	(1–) Many	1–2	1	1 (−2)

## Data Availability

The sequences produced are available in GenBank (accession numbers available in Table S3 in the Supporting Information), the public repository of NCBI. Other supporting data are included as Supplementary Files.

## Supplementary Materials

The following supporting information can be downloaded at https://www.mdpi.com/article/10.3390/plants15213437/s1: Table S1: Characteristics of each of the molecular markers, individually and combined, as well as the nucleotide substitution models used; Table S2: Voucher information of the collected specimens used in the phylogenetic analyses; Table S3: GenBank accessions used in the analyses; Figure S1: Phylogenetic relationships among *Waltheria*, *Melochia*, and *Stegowaltheria* produced with Bayesian inference of nuclear rITS (50% majority rule consensus tree). Posterior probabilities (≥0.50) are listed above the branches, and bootstrap percentages from maximum likelihood (≥50) are indicated below the branches; Figure S2: Phylogenetic relationships among *Waltheria*, *Melochia*, and *Stegowaltheria* produced using Bayesian inference of combined plastid regions (*matK* exon and *trnL-F* intron and spacer) (50% majority rule consensus tree). Posterior probabilities (≥0.50) are listed above the branches, and bootstrap percentages from maximum likelihood (≥50) are indicated below the branches; Figure S3: Phylogenetic relationships among *Waltheria*, *Melochia*, and *Stegowaltheria* produced using maximum likelihood of one nuclear (rITS) and two plastid regions (*matK* exon and *trnL-F* intron and spacer) (50% majority rule consensus tree). Bootstrap percentages (≥50) are indicated above the branches.

## Abbreviations

The following abbreviations are used in this manuscript:

PPs	Posterior Probabilities
BPs	Bootstrap Percentages

## References

[1] Colli-Silva M., Pérez-Escobar O.A., Ferreira C.D., Costa M.T., Gerace S., Coutinho T.S., Yoshikawa V.N., Antonio-Domingues H., Hernández-Gutiérrez R., Bovini M.G. (2025). Taxonomy in the light of incongruence: An updated classification of Malvales and Malvaceae based on phylogenomic data. Taxon.

[2] Whitlock B.A., Bayer C., Baum D.A. (2001). Phylogenetic relationships and floral evolution of the Byttnerioideae (“Sterculiaceae” or Malvaceae s.l.) based on sequences of the chloroplast gene. ndhF. Syst. Bot..

[3] Schumann K., Engler A., Prantl K. (1890). Sterculiaceae. Die Natürlichen Pflanzenfamilien.

[4] Saunders J.G. (1993). Four new distylous species of *Waltheria* (Sterculiaceae) and a key to the Mexican and Central American species and species groups. Syst. Bot..

[5] Coutinho T.S. (2021). Taxonomia de *Waltheria* L. (Byttnerioideae, Malvaceae) no Brasil. Ph.D. Thesis.

[6] Coutinho T.S., Colli-Silva M., Pirani J.R. (2026). *Waltheria* in Flora e Funga do Brasil. Jardim Botânico do Rio de Janeiro. https://floradobrasil.jbrj.gov.br/FB9270.

[7] Schumann K., Martius C.F.P., Eichler A.W., Urban I. (1886). Waltheria. Flora Brasiliensis.

[8] Rose J.N., Davenport G.E. (1899). Studies of Mexican and Central American plants—No. 2. Contr. U.S. Natl. Herb..

[9] Saunders J.G. (1995). Systematics and Evolution of *Waltheria* (Sterculiaceae, Hermannieae). Ph.D. Thesis.

[10] Saunders J.G. (2021). Five new clavate-stigma *Waltheria* species endemic to Brazilian Cerrado (Malvaceae s.l., Byttnerioideae, Hermannieae). Darwiniana.

[11] Richardson J.E., Whitlock B.A., Meerow A.W., Madriñán S. (2015). The age of chocolate: A diversification history of *Theobroma* and Malvaceae. Front. Ecol. Evol..

[12] Coutinho T.S., Sader M.A., Pedrosa-Harand A., Alves M. (2022). *Waltheria marielleae* (Byttnerioideae, Malvaceae), a new species from north-eastern Brazil supported by morphological and phylogenetic evidence. Plant Ecol. Evol..

[13] Dorr L.J., Barnett L.C. (1989). A revision of *Melochia* section *Physodium* (Sterculiaceae) from Mexico. Brittonia.

[14] Goldberg A. (1967). The genus *Melochia* L. (Sterculiaceae). Contr. U.S. Natl. Herb..

[15] Coutinho T.S., Alves M. (2020). *Waltheria glabribracteata* (Byttnerioideae, Malvaceae), a new species with elongate-plumose stigmas from South America. Phytotaxa.

[16] Pessoa E.M., Nollet F., Magalhães R.F., Viruel J., Pinheiro F., Chase M.W. (2022). Nuclear–plastid discordance indicates past introgression in *Epidendrum* species (Laeliinae: Orchidaceae) with highly variable chromosome numbers. Bot. J. Linn. Soc..

[17] Coutinho T.S., Colli-Silva M., Alves M. (2020). Novelties in Brazilian *Waltheria* L. (Byttnerioideae, Malvaceae): Two new species and one re-establishment. Acta Botan. Bras..

[18] Doyle J.J., Doyle J.L. (1987). A rapid DNA isolation procedure form small quantities of fresh leaf tissue. Phytochem. Bull..

[19] Johnson L.A., Soltis D.E. (1994). MatK DNA sequences and phylogenetic reconstruction in Saxifragaceae s. str. Syst. Bot..

[20] Nyffeler R., Bayer C., Alverson W.S., Yen A., Whitlock B.A., Chase M.W., Baum D.A. (2005). Phylogenetic analysis of the Malvadendrina clade (Malvaceae s.l.) based on plastid DNA sequences. Org. Divers. Evol..

[21] Taberlet P., Gielly L., Pautou G., Bouvet J. (1991). Universal primers for amplification of three noncoding regions of chloroplast DNA. Plant Mol. Biol..

[22] Sun Y., Skinner D.Z., Liang G.H., Hulbert S.H. (1994). Phylogenetic analysis of Sorghum and related taxa using internal transcribed spacers of nuclear ribosomal DNA. Theor. Appl. Genet..

[23] Edgar R.C. (2004). MUSCLE: Multiple sequence alignment with high accuracy and high throughput. Nucleic Acids Res..

[24] Farris J.S., Kallersjo M., Kluge A.G., Bult C. (1995). Constructing a significance test for incongruence. Syst. Biol..

[25] Swofford D.L. (2002). PAUP*: Phylogenetic Analysis Using Parsimony (* and Other Methods).

[26] Darriba D., Taboada G.L., Doallo R., Posada D. (2012). JModelTest 2: More models, new heuristics and parallel computing. Nat. Methods.

[27] Ronquist F., Teslenko M., Van der Mark P., Ayres D.L., Darling A., Höhna S., Larget B., Liu L., Suchard M.A., Huelsenbeck J.P. (2012). MrBayes 3.2: Efficient Bayesian phylogenetic inference and model choice across a large model space. Syst. Biol..

[28] Miller M., Holder M., Vos R., Midford P.E., Liebowitz T., Chan L., Hoover P., Warnow T. (2010). The CIPRES Portals. http://www.phylo.org/sub_sections/portal.

[29] Rambaut A., Drummond A.J., Xie D., Baele G., Suchard M.A. (2018). Posterior summarization in Bayesian phylogenetics using Tracer 1.7. Syst. Biololy.

[30] Stamatakis A. (2014). Raxml version 8: A tool for phylogenetic analysis and post-analysis of large phylogenies. Bioinformatics.

[31] Rambaut A. (2018). FigTree v1.4.4, 2018. http://tree.bio.ed.ac.uk/software/figtree/.

[32] Cummings M.P., Handley S.A., Myers D.S., Reed D.L., Rokas A., Winka K. (2003). Comparing bootstrap and posterior probability values in the four-taxon case. Syst. Biololy.

[33] Erixon P., Svennblad B., Britton T., Oxelman B. (2003). Reliability of Bayesian posterior probabilities and bootstrap frequencies in phylogenetics. Syst. Biol..

[34] Simmons M.P., Pickett K.M., Miya M. (2004). How meaningful are Bayesian support values?. Mol. Biol. Evol..

