# Supplementary material for: Molecular Phylogeny of Waltheria L. (Malvaceae, Byttnerioideae) Supports the Recognition of Stegowaltheria as a Distinct Genus
Source: Plants (Basel). 2026 Nov 4;15(21):3437. doi: 10.3390/plants15213437 (PMC13766056; doi:10.3390/plants15213437)
Supplement: Supplementary file 1 [file plants-15-03437-s001.zip › Figure S1.pdf]

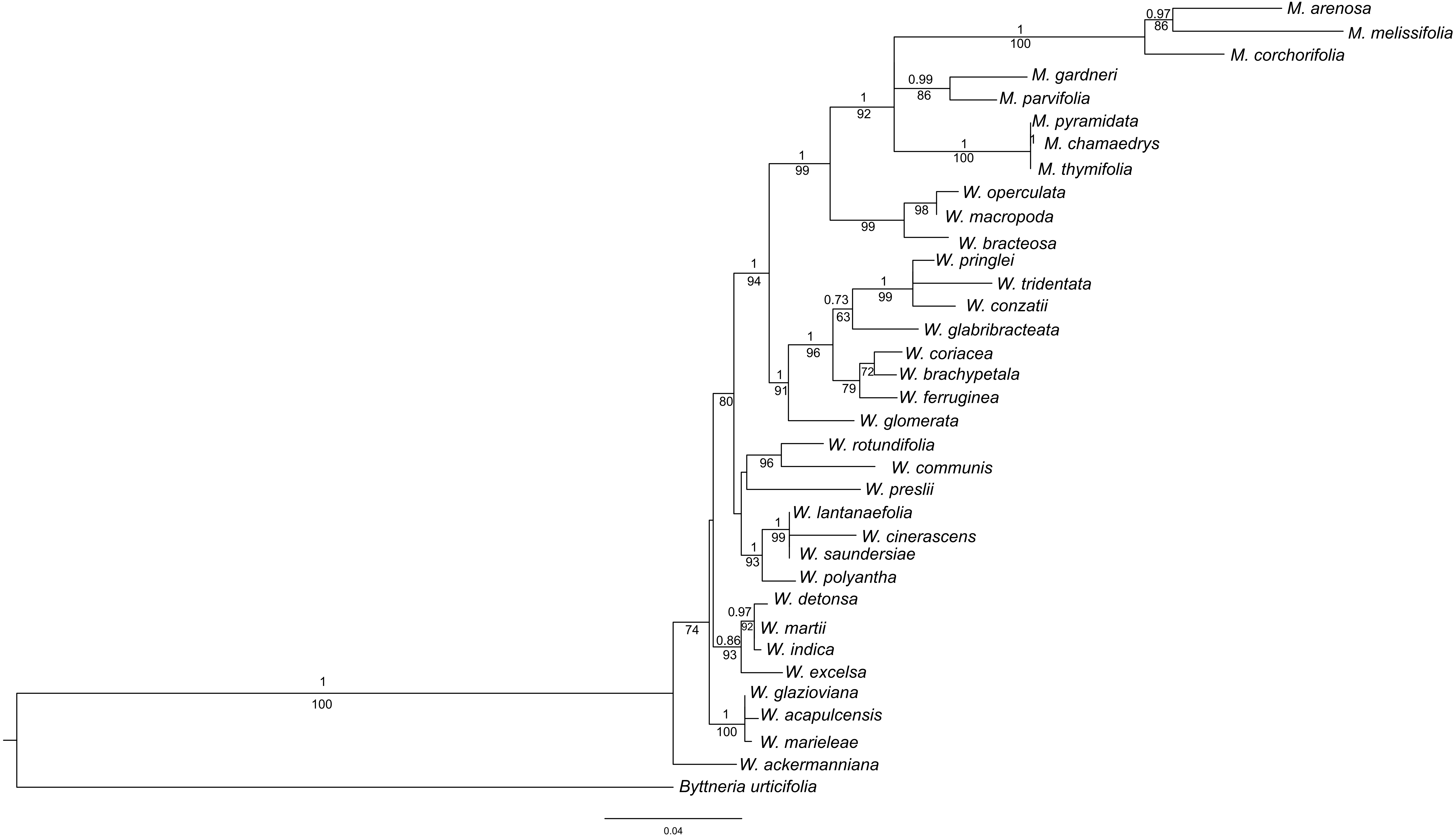
M. arenosa
M. melissifolia
M. corchorifolia
M. gardneri
M. parvifolia
M. pyramidata
M. chamaedrys
M. thymifolia
W. operculata
W. macropoda
W. bracteosa
W. pringlei
W. tridentata
W. conzatii
W. glabribracteata
W. coriacea
W. brachypetala
W. ferruginea
W. glomerata
W. rotundifolia
W. communis
W. preslii
W. lantanaefolia
W. cinerascens
W. saundersiae
W. polyantha
W. detonsa
W. martii
W. indica
W. excelsa
W. glazioviana
W. acapulcensis
W. marieleae
W. ackermanniana
Byttneria urticifolia
0.04
