# Supplementary material for: Molecular Phylogeny of Waltheria L. (Malvaceae, Byttnerioideae) Supports the Recognition of Stegowaltheria as a Distinct Genus
Source: Plants (Basel). 2026 Nov 4;15(21):3437. doi: 10.3390/plants15213437 (PMC13766056; doi:10.3390/plants15213437)
Supplement: Supplementary file 1 [file plants-15-03437-s001.zip › Table S1.pdf]

Table S1. Characteristics of each molecular marker, individually and combined, as well as nucleotide substitution models used.

	ITS	*matK*	*trnLF*	Plastid combined	All combined
**Taxon number**	35	30	30	41	41
**Alignment length**	545	806	1097	1903	2448
**Number of variable positions**	217	114	112	226	443
**Number of parsimony informative sites**	122	32	26	58	180
**Substitution model**	GTR+G	TVM+G	GTR+G	-	-
