# Supplementary material for: Molecular Phylogeny of Waltheria L. (Malvaceae, Byttnerioideae) Supports the Recognition of Stegowaltheria as a Distinct Genus
Source: Plants (Basel). 2026 Nov 4;15(21):3437. doi: 10.3390/plants15213437 (PMC13766056; doi:10.3390/plants15213437)
Supplement: Supplementary file 1 [file plants-15-03437-s001.zip › Table S2.pdf]

Table S2. Voucher information of the collected specimens used in the phylogenetic analyses.

Species	Locality	Collector name and number (herbarium)
Melochia arenosa Benth.	Brazil, Mato Grosso	*T.S. Coutinho et al. 739* (UFMT)
Melochia gardneri Sprague	Brazil, Goiás	*M. Figueira 1165* (CEN)
Melochia melissifolia Benth.	Brazil, Mato Grosso	*T.S. Coutinho et al. 742* (UFMT)
Melochia parvifolia Kunth	Brazil, Mato Grosso	*T.S. Coutinho 614* (UFMT)
Melochia pyramidata L.	Brazil, Mato Grosso	*T.S. Coutinho 736* (UFMT)
Melochia spicata (L.) Fryxell	Brazil, Mato Grosso	*T.S. Coutinho et al. 759* (UFMT)
Waltheria acapulcensis Rose	Mexico, Oaxaca	*E. Martínez S. & J. Castrejón 33008* (MEXU)
Waltheria ackermanniana K. Schum.	Brazil, Pernambuco	*T.S. Coutinho et al. 335* (UFP)
Waltheria acuminata	Mexico, Nayarit	*O. Tellez et al. 12199* (MEXU)
Waltheria bicolor J.G. Saunders	Mexico, Colima	*A.C. Sanders et al. 11753* (MEXU)
Waltheria brachypetala Turcz.	Brazil, Pernambuco	*T.S. Coutinho et al. 336* (UFP)
Waltheria bracteosa A. St.-Hil. & Naudin	Brazil, Bahia	*F. Gomes-Silva 635* (HUEFS)
Waltheria cinerascens A. St.-Hil.	Brazil, Bahia	*T.S. Coutinho et al. 232* (CEN)
Waltheria communis A. St.-Hil.	Brazil, Distrito Federal	*T.S. Coutinho & C. Alcântara 661* (UFMT)
Waltheria conzatii Standl.	Mexico	*S.M. Bende 565* (MEXU)
Waltheria detonsa A. Gray	Mexico	*J. Agundez 602* (MEXU)
Waltheria excelsa Turcz.	Brazil, Tocantins	*B. Schindler 387* (CEN)
Waltheria ferruginea A. St.-Hil.	Brazil, Minas Gerais	*D. Zavatin et al. 1676*(SPF)
Waltheria fryxellii J.G. Saunders	Mexico, Guerrero	*J.C. Soto 12912* (MEXU)
Waltheria glabribracteata T.S. Cot. & M. Alves	Brazil, Mato Grosso	*T.S. Coutinho 683* (UFMT)
Waltheria glazioviana K. Schum.	Brazil, Espírito Santo	*G. Antar et al. 4547* (SAMES)
Waltheria glomerata C. presl.	Brazil, Mato Grosso	*T.S. Coutinho & M. Zappani 515* (UFMT)
Waltheria lundelliana J.G. Saunders	Mexico, Nayarit	*J.I. Calzada et al. 19124* (MEXU)
Waltheria macropoda Turcz.	Brazil, Ceará	*T.S. Coutinho et al. 623* (HCDAL)
Waltheria marielleae T.S. Cout. & M. Alves	Brazil, Alagoas	*T.S. Coutinho et al. 375* (UFP)
Waltheria martii Colla	Brazil, Ceará	*T.S. Coutinho et al. 625* (HCDAL)
Waltheria operculata Rose	Brazil, Ceará	*T.S. Coutinho et al. 622* (HCDAL)
Waltheria preslii Walp.	Mexico	*R.G. Sosa 456* (MEXU)

Waltheria pringlei Rose & Standl.	Mexico	*M. Martínez 2792* (MEXU)
Waltheria rotundifolia Schrank	Brazil, Ceará	*T.S. Coutinho 621* (HCDAL)
Waltheria saundersiae T.S. Cout. & M. Alves	Brazil, Bahia	*T.S. Coutinho et al. 238* (UFP)
Waltheria tridentata J.G. Saunders	Mexico, Oaxaca	*L.A.H. Pérez & A. Torres 405* (MEXU)
